# A systemic review and an updated meta-analysis: minimally invasive vs open pancreaticoduodenectomy

**DOI:** 10.1038/s41598-017-02488-4

**Published:** 2017-05-22

**Authors:** Zhanwei Zhao, Zifang Yin, Zhenning Hang, Gang Ji, Quanxin Feng, Qingchuan Zhao

**Affiliations:** 1 Xijing Hospital of Digestive Diseases, the Fourth Military Medical University, 127 Changle Western Road, Xi’an, China; 2 Shaanxi Maternal and Child Health Hospital, Xi’an, China

## Abstract

The feasible of minimally invasive pancreaticoduodenectomy (MIPD) remains controversial when compared with open pancreaticoduodenectomy (OPD). We conducted a systemic review and meta-analysis to summarise the available evidence to compare MIPD vs OPD. We systemically searched PubMed, EMBASE and Web of Science for studies published through February 2016. The primary endpoint was postoperative pancreatic fistula (POPF, grade B/C). A total of 27 studies involving 14,231 patients (2,377 MIPD and 11,854 OPD) were included. MIPD was associated with longer operative times (*P* < 0.01) and increased mortality (*P* < 0.01), but decreased estimated blood loss (*P* < 0.01), decreased delayed gastric emptying (*P* < 0.01), increased R0 resection rate (*P* < 0.01), decreased wound infection (*P* = 0.03) and shorter hospital stays (*P* < 0.01). There were no significant differences in BMI (*P* = 0.43), tumor size (*P* = 0.17), lymph nodes harvest (*P* = 0.57), POPF (*P* = 0.84), reoperation (*P* = 0.25) and 5-year survival rates (*P* = 0.82) for MIPD compared with OPD. Although there was an increased operative cost (*P* < 0.01) for MIPD compared with OPD, the postoperative cost was less (*P* < 0.01) with the similar total costs (*P* = 0.28). MIPD can be a reasonable alternative to OPD with the potential advantage of being minimally invasive. However, MIPD should be performed in high-volume centers and more randomized-controlled trials are needed to evaluate the appropriate indications of MIPD.

## Introduction

Minimally invasive pancreaticoduodenectomy (MIPD), including laparoscopic pancreaticoduodenectomy (LPD) and robotic pancreaticoduodenectomy (RPD), is considered to be one of the most challenging laparoscopic surgeries. LPD was first described by Gagner and Pomp in 1994^1^. Some reports have suggested that the laparoscopic procedure was not only disappointing and without benefit compared with OPD, but impossible to gain adequate margins for cancers^2, 3^. The first case of RPD was reported in 2003 by Giulianotti *et al*.^4^, and feasibility and safety of RPD were unclear when compared with OPD due to the longer operative times and other disadvantages, especially with respect to radical resection, which is associated with the patient’ s prognosis^5, 6^.

The early stages of LPD and RPD development were uncertain due to the complex procedures, high conversion rate, long operative time and the questions associated with radical tumor resection. However, in recent years, additional studies have reported that LPD is safe and technically feasible^7^. Kendrick *et al*.^3^ reported 108 cases of total laparoscopic pancreaticoduodenectomy (TLPD) and demonstrated that TLPD was not only safe and feasible but also had the advantages of faster postoperative recovery, shorter hospital stays and allowing the patients to pursue adjuvant treatment options. In addition, RPD, with its potential advantages such as decreased numbers of complications and surgical site infections and reduced length of stay in the intensive care unit, has also been recognized by pancreatic surgeons^8, 9^. Nevertheless, the technical difficulties, complications, a high conversion rate, radical tumor resection and high costs of MIPD are still controversial^10, 11^.

Thus, we conducted a systematic analysis based on more sufficient evidence and a quantitative synthesis of the eligible data with the following objectives: (1) to provide an update with more sufficient evidence published up to February 2016 on the comparison between MIPD and OPD; (2) to further examine the comparison between the type of MIPD and OPD according to subgroup analyses including the type of MIPD, geographic area, sample size, publication year and quality score; and (3) to examine the cost and 5-year survival rate to estimate the burden of cost and long-term survival rate associated with MIPD compared with OPD.

## Methods

### Search strategy

Publications were identified using a search strategy in PubMed, EMBASE and Web of Science. The last studies were searched for in February 2016. The following search terms were used. For minimally invasive techniques, the search keywords were *“laparoscopic”, “laparoscopy”, “robot”, “robotic”, “minimal invasive”, “minimally invasive”* and *“da Vinci”*. For procedures, the search keywords were *“pancreaticoduodenectomy”, “pancreatoduodenectomy”, “duodenopancreatectomy”, “whipple”, “pancreatectomy”, “pancreas”* and *“pancreatic”*. The reference lists in the included studies were also searched manually to identify additional literature. The two sets of keywords were combined individually, and the eligibility criteria were independently judged by two of the authors (Zhanwei Zhao and Zifang Yin). Only English language studies were considered. The studies were limited only to those performed in humans.

### Selection criteria

1. Studies unrelated to our topics were excluded.
2. Case-control studies and cohort studies were included.
3. Case reports without comparison with OPD were excluded.
4. Data that were incomplete or could not be combined were excluded.
5. Narrative reviews, systematic reviews and meta-analyses were excluded.
6. Cases in which only comments, editorials, letters and the abstract could be obtained were excluded.
7. Studies with a total case number less than 10 were excluded.
8. The most recent studies, studies with the most samples, and the highest quality studies that included reports with the same patients were selected.
9. Laparoscopic or robotic pancreatectomy without pancreaticoduodenectomy was excluded.

### Study quality

Study quality in this meta-analysis was assessed using the Newcastle-Ottawa Scale (NOS)^12^. The NOS was judged in three parts, including the elucidation of exposure or the outcomes of interest for case-control or cohort studies, the selection of the study populations and the comparability of the populations. Two of the authors (Zhanwei Zhao and Zifang Yin) independently assessed the quality of the studies, and a consensus decision was made regarding any discrepancies in interpretations by the third author (Qingchuan Zhao). The range of the NOS is 0–9 stars, and high quality studies are those that receive 7 or more stars^13^.

### Data Extraction

A data extraction sheet was established to enter the data from each study, including the first author, year of publication, country, study type, study period, study centers, study population, cancer diagnosis, type of MIPD, mortality and NOS score (Table 1).

**Table 1:** Characteristics of included studies.

First author	Year	Country	Study type	Study period	Center performing	Study populationMIPD/OPD	Cancers MIPD/OPD	Type of MIPD	Mortality (n) MIPD/OPD	NOS score
Cho^30^	2009	Japan	Retrospective	2007–2008	1	15/15	8/15	LPD (LPPPD)	0/0	7
Buchs^31^	2011	USA	Retrospective	2002–2010	1	44/39	33/27	RLPD	2/1	7
Zhou^28^	2011	China	Retrospective	2009 Jan–Dec	1	8/8	8/8	RLPD	0/1	6
Zureikat^27^	2011	USA	Retrospective	2008–2010	1	14/14	13/12	LPD	7/0	7
Asbun^32^	2012	USA	Retrospective	2005–2011	1	53/215	51/195	LPD/TLPD/LPPPD	3/19	9
Chalikonda^33^	2012	USA	Retrospective	2009–2010	1	30/30	18/18	RLPD	1/0	8
Kuroki^34^	2012	Japan	Retrospective	2008–2010	1	20/31	20/30	LPD/LPPPD/LSSPPD	NR	5
Lai^5^	2012	China	Retrospective	2000–2012	1	20/67	15/53	RLPD	0/3	6
Mesleh^26^	2013	USA	Retrospective	2009–2012	1	75/48	73/42	LPD	NR	6
Bao^8^	2014	USA	Retrospective	2009–2011	1	28/28	19/26	LPD	2/2	7
Croome^3^	2014	USA	Retrospective	2008–2013	1	108/214	NR	TLPD	1/4	9
Hakeem^29^	2014	UK	Retrospective	2005–2009	1	12/12	12/12	LPD	3/6	6
Langan^24^	2014	USA	Retrospective	2010–2013	1	28/25	16/16	LPD	NR	6
Speicher^21^	2014	USA	Retrospective	2010–2013	1	56/84	45/62	LPD/TLPD	3/6	8
Wang^35^	2014	Canada	Retrospective	2009–2013	1	13/20	10/15	LPD	1/4	7
Wellner^36^	2014	Germany	Retrospective	1996–2013	2	40/40	28/28	LPD (LPPPD)	0/1	7
Adam^19^	2015	USA	Retrospective	2010–2011	NR	983/6078	831/5234	LPD	50/188	7
Baker^9^	2015	USA	Retrospective	2012–2013	1	22/49	18/40	RLPD	0/2	7
Chen^6^	2015	China	Prospective	2010–2013	1	60/120	38/76	RLPD	1/3	8
Dokmak^23^	2015	France	Retrospective	2011–2014	1	46/46	40/38	LPD	1/0	8
Liang^37^	2015	Canada	Retrospective	2011–2013	1	15/29	9/23	LPD/TLPD	1/1	6
Mendoza^38^	2015	Korea	Retrospective	2014 Jun–Dec	1	18/34	14/31	LPD	NR	5
Senthilnathan^25^	2015	India	Retrospective	2006–2011	1	45/118	NR	LPD/LPPPD	NR	6
Sharpe^20^	2015	USA	Retrospective	2010–2011	9	384/4037	NR	LPD	20/150	7
Song^39^	2015	Korea	Retrospective	2007–2012	1	97/198	93/167	LPD (LPPPD)	0/0	8
Tan^40^	2015	China	Retrospective	2009–2014	1	30/30	27/26	TLPD	0/1	6
Tee^41^	2015	USA	Retrospective	2007–2014	1	113/225	75/192	LPD/TLPD/LPPPD	5/3	7

MIPD: minimally invasive pancreaticoduodenectomy; OPD: open pancreaticoduodenectomy; LPD: laparoscopic pancreaticoduodenectomy; TLPD: total laparoscopic pancreaticoduodenectomy; LPPPD: laparoscopic pylorus-preserving pancreaticoduodenectomy; LSSPPD: laparoscopic subtotal stomach-preserving pancreaticoduodenectomy; RLPD: robotic-assisted laparoscopic pancreaticoduodenectomy; NOS: Newcastle-Ottawa Scale; NR: no record.

### Statistical analysis

The data were collected and extracted using SPSS 17.0 (Chicago, Illinois, USA). RevMan5.3 (The Cochrane Collaboration, Oxford, UK) and STATA version 12.1 (STATA Corporation, College Station, TX) software were used for the data synthesis and analysis.

Random-effects models were used to estimate the pooled results. Weighted mean differences (WMD, for continuous data) and odds ratios (OR, for event-related data) were used to express the estimates. Additionally, all reports with their corresponding 95% confidence intervals (CIs) were calculated.

Heterogeneity among studies was detected using Q (a *P* < 0.1 was considered representative of statistically significant heterogeneity) and *I* ^2^ statistics (25%, 50% and 75% indicated low, moderate and high heterogeneity, respectively. In this analysis, *I* ^2^ < 50% indicated low heterogeneity, and *I* ^2^ > 50% indicated substantial heterogeneity)^14^. Subgroup analyses were conducted to further explore the sources of heterogeneity by type of MIPD, geographic area, sample size, publication year and quality score (*P* < 0.1 was considered to be a significant source of heterogeneity).

The publication bias was assessed using funnel plots and Egger’s test (*P* < 0.1 was considered to be a significant publication bias)^15, 16^. Sensitivity analyses were conducted to investigate the influence of a specific study on the pooled risk estimate by removing one study in each turn.

## Results

### LPD RLPD

#### Literature selection, study characteristics and quality scores

Supplementary Figure 1 shows a flowchart of the search strategy that was used to select the eligible studies. A total of 4,442 studies were initially identified for this meta-analysis: 2,039 studies were excluded for duplication, and 2,403 studies were selected for further consideration. Of those, 2,276 studies were excluded after reviewing the titles and abstracts, and 101 studies were excluded after reviewing the full-text articles. Finally, 27 studies met the eligibility criteria after the addition of one study from the reference review.

The 27 selected studies were conducted in 9 countries worldwide and included 21 studies of LPD and 6 studies of RPD (all of them were robot-assisted pancreaticoduodenectomy, RLPD). The Quality Assessment of Diagnostic Accuracy studies (QUADAS) tool was used to assess the quality of the included studies. Generally, the eligible studies met most of the criteria, and the quality scores ranged from 5 to 9 (Table 1).

#### Preoperative outcomes

The results of the patient characteristics in the MIPD and OPD groups, including age, sex, body mass index (BMI), American Society of Anesthesiologists (ASA), tumor size and cancer diagnosis, are shown in Table 2 and Supplementary Figure 2. The patients were comparable in age (WMD = 1.44 years, 95% = −0.60–3.48 years, *P* = 0.17), gender (OR = 0.99, 95% = 0.76–1.30, *P* = 0.94), BMI (WMD = −0.31 kg/m^2^, 95% CI = −1.09–0.46 kg/m^2^, *P* = 0.43), ASA (WMD = 0.03, 95% CI = −0.60–0.67, *P* = 0.92), tumor size (WMD = −0.06 cm, 95% CI = −0.15–0.03 cm, *P* = 0.17) and cancer diagnosis (OR = 0.95, 95% = 0.70–1.30, *P* = 0.76).

**Table 2:** Meta-analysis of different outcomes variables.

Variable	Included studies	Patients (n) MIPD/OPD	OR/WMD	95% CI	*P* value	*P*_h_	*I* ^*2*^ (%)
Age (year)	18	2052/11483	1.44	−0.60, 3.48	0.17	<0.01	90
Sex (male)	26	1993/10817	0.99	0.76, 1.30	0.94	<0.01	71
ASA score	2	59/54	0.03	−0.60, 0.67	0.92	<0.01	89
Cancers	22	1840/7485	0.95	0.70, 1.30	0.76	<0.01	51
Tumor size (cm)	14	1854/11068	−0.06	−0.15, 0.03	0.17	0.02	49
Mortality	21	2068/11600	1.42	1.13, 1.80	**0.003**	0.53	0
BMI (Kg/m^2^)	16	703/1195	−0.31	−1.09, 0.46	0.43	<0.01	77
Operative time (min)	17	687/1174	67.37	25.11, 109.63	**0.002**	<0.01	95
Estimated blood loss (mL)	14	597/1061	−324.47	−492.37, −156.57	**0.0002**	<0.01	93
R0	19	749/4195	1.40	1.15, 1.70	<**0.001**	0.94	0
Lymph node harvest	17	896/5204	0.36	−0.88, 1.61	0.57	<0.01	73
POPF	23	933/1491	0.98	0.77, 1.24	0.84	0.96	0
Delayed gastric emptying	18	814/1330	0.64	0.48, 0.84	**0.002**	0.84	0
Wound infection	14	520/958	0.71	0.53, 0.96	**0.03**	0.07	38
Length of hospital stay (days)	15	948/5005	−3.14	−4.42, −1.87	<**0.01**	<0.01	69
Re-operation	13	518/873	0.78	0.51, 1.19	0.25	0.45	0
Operative cost ($)	3	147/295	6663.75	2079.60, 11247.91	**0.004**	<0.01	99
Postoperative cost ($)	4	124/220	−550.76	−652.24 −449.29	<**0.001**	0.32	14
Total cost ($)	4	213/428	3018.56	−3359.60, 9396.72	0.35	<0.01	88
5-year survival	3	142/709	1.14	0.37, 3.46	0.82	0.66	0

*P* value: P value for the overall effect; *P*_h_: *P* value for heterogeneity; ASA: American Society of Anesthesiologists; BMI: body mass index (Kg/m^2^); POPF: clinically relevant postoperative pancreatic fistula; OR: odds ratio; WMD: weight mean difference. Bold text indicates statistical significance.

Subgroup analysis (Supplementary Figure 2) of LPD vs OPD and RLPD vs OPD suggested that there were no differences between LPD and OPD in age (WMD = 0.95 year, 95% = −1.33–3.24 years, *P* = 0.41), gender (OR = 0.94, 95% = 0.68–1.31, *P* = 0.72), BMI (WMD = −0.58 kg/m^2^, 95% CI = −1.40–0.23 kg/m^2^, *P* = 0.16), tumor size (WMD = −0.05 cm, 95% CI = −0.14–0.05 cm, *P* = 0.33) or cancer diagnosis (OR = 0.94, 95% = 0.62–1.43, *P* = 0.76); there were no differences between RLPD and OPD in age (WMD = 3.19 years, 95% = −0.26–6.64 years, *P* = 0.07), gender (OR = 1.17, 95% = 0.80–1.71, *P* = 0.42), BMI (WMD = 0.78 kg/m^2^, 95% CI = −1.07–2.63 kg/m^2^, *P* = 0.41), tumor size (WMD = −0.28 cm, 95% CI = −0.62–0.07 cm, *P* = 0.11) and cancer diagnosis (OR = 1.03, 95% = 0.68–1.56, *P* = 0.90). There was no subgroup analysis for the ASA score because of the limited amount of data.

#### Intraoperative outcomes

The results of intraoperative outcomes in the MIPD and OPD groups, including the operative time, estimated blood loss, R0 and lymph node harvest, are shown in Table 2 and Supplementary Figure 3. The results showed that the operative times were longer for MIPD (WMD = 67.37 minutes, 95% CI = 25.11–109.63 minutes, *P* < 0.01), but that there was less estimated blood loss (WMD = −324.47 mL, 95% CI = −492.37, −156.57 mL, *P* < 0.01) and an increased R0 resection rate (OR = 1.40, 95% CI = 1.15–1.70, *P* < 0.01) with no significant differences in lymph nodes harvest (WMD = 0.36, 95% CI = −0.81–1.61, *P* = 0.57).

The subgroup analysis (Supplementary Figure 3) of LPD vs OPD and RLPD vs OPD suggested that there were increased operative times for LPD (WMD = 60.25 minutes, 95% CI = 17.16–103.34 minutes, *P* < 0.01) but no difference for RLPD (WMD = 95.51 minutes, 95% CI = −42.24–233.27 minutes, *P* = 0.17); decreased estimated blood loss for LPD (WMD = −341.61 mL, 95% CI = −578.57, −104.65 mL, *P* < 0.01) but no difference for RLPD (WMD = −289.81 mL, 95% CI = −585.27, 5.66 mL, *P* = 0.05); and no difference of R0 resection rate for RLPD (OR = 1.85, 95% CI = 0.94–3.64, *P* = 0.08) but an increased R0 resection rate for LPD (OR = 1.37, 95% CI = 1.11–1.67, *P* < 0.01). There were no differences in lymph node harvests between LPD (WMD = −0.09, 95% CI = −1.50–1.31, *P* = 0.90) and RLPD (WMD = 2.13, 95% CI = −0.91–5.17, *P* = 0.17).

#### Postoperative outcomes

The results of the postoperative outcomes of MIPD and OPD groups, including mortality, postoperative pancreatic fistula (POPF, defined by International Study Group on Pancreatic Fistula)^17^, delayed gastric emptying (defined by International Study Group of Pancreatic Surgery)^18^, wound infection, overall length of hospital stay and reoperation, are shown in Table 2 and Supplementary Figure 4. The results showed that in the MIPD group there was increased mortality (OR = 1.42, 95% CI = 1.09–1.86, *P* = 0.01), but shorter hospital stays (WMD = −3.14 days, 95% CI = −4.42, −1.87 days, *P* < 0.01), decreased delayed gastric emptying (OR = 0.64, 95% CI = 0.48–0.84, *P* < 0.01) and decreased wound infection (OR = 0.71, 95% CI = 0.53–0.96, *P* = 0.03) with no significant differences in POPF (OR = 0.98, 95% CI = 0.77–1.24, *P* = 0.84) and reoperation (OR = 0.78, 95% CI = 0.51–1.19, *P* = 0.25).

The subgroup analysis (Supplementary Figure 4) of LPD vs OPD and RLPD vs OPD suggested that there were shorter hospital stays associated with LPD (WMD = −2.47 days, 95% CI = −3.77, −1.17 days, *P* < 0.01) and RLPD (WMD = −5.46 days, 95% CI = −8.86, −2.07 days, *P* < 0.01); decreased delayed gastric emptying for LPD (OR = 0.67, 95% CI = 0.49–0.90, *P* < 0.01) with no difference for the RLPD group (OR = 0.52, 95% CI = 0.26–1.04, *P* = 0.06); decreased wound infection for RLPD (OR = 0.18, 95% CI = 0.06–0.53, *P* < 0.01) with no difference for the LPD group (OR = 0.81, 95% CI = 0.51–1.30, *P* = 0.38); and high mortality for LPD (OR = 1.46, 95% CI = 1.15–1.85, *P* < 0.01) with no difference for the RLPD group (OR = 0.89, 95% CI = 0.29–2.70, *P* = 0.84). There were no differences in POPF for LPD (OR = 1.01, 95% CI = 0.77–1.32, *P* = 0.95) or RLPD (OR = 0.85, 95% CI = 0.50–1.46, *P* = 0.55) and no differences in reoperation between LPD (OR = 0.93, 95% CI = 0.57–1.51, *P* = 0.76) and RLPD (OR = 0.51, 95% CI = 0.22–1.15, *P* = 0.10).

#### Cost and long survival rate

Additionally, we further examined the operative cost, postoperative cost, total cost and 5-year survival rate to estimate the patients’ cost and length of survival, which are shown in Table 2 and Supplementary Figure 5. The meta-analysis showed that although there was increased operative cost (WMD = $6663.75, 95% CI = $2079.60 to $11247.91, *P* < 0.01) with high heterogeneity (*P* < 0.01, I^2^ = 99%) for MIPD compared with OPD, the postoperative cost was less (WMD = −$550.76, 95% CI = −$652.24 to −$499.29, *P* < 0.01) without significant heterogeneity (*P* = 0.32, I^2^ = 14%), and the total cost was similar between the two groups (*P* = 0.28). This analysis suggested that the pooled 5-year survival rate was similar following the two procedures (OR = 1.14, 95% CI = 0.37–3.46, *P* = 0.82) without significant heterogeneity (*P* = 0.66, I^2^ = 0%).

#### Heterogeneity and publication bias

The primary endpoint was postoperative pancreatic fistula (International Study Group on Pancreatic Fistula grade B/C). Random-effects models were used to estimate the pooled results. This analysis suggested that there was no significant heterogeneity in the results between MIPD and OPD (*P* = 0.96, I^2^ = 0%). Subgroup analyses were conducted to further explore potential heterogeneity, and the results (Table 3) showed that the differences in ORs were not significant (*P* > 0.05, I^2^ = 0%) for the type of MIPD, geographic area, sample size, publication year or NOS score.

**Table 3:** Subgroup analyses of MIPD vs OPD.

	n	OR (95% CI)	*P*_s_	*I*_*s*_^2^ (%)	*P*_t_
**All studies**	23	0.98 (0.77–1.24)	0.96	0	0.84
**Type of MIPD**					
LPD	17	1.01 (0.77–1.32)	0.98	0	0.95
RLPD	6	0.85 (0.50–1.46)	0.38	5	0.55
**Geographic area**					
Europe	3	1.11 (0.59–2.08)	0.28	21	0.75
America	12	0.97 (0.72–1.31)	0.97	0	0.85
Asia	8	0.91 (0.55–1.51)	0.61	0	0.72
**Sample size**					
<100	16	1.01 (0.70–1.45)	0.89	0	0.98
≥100	7	0.95 (0.70–1.30)	0.79	0	0.77
**Publication year**					
Before 2014	9	1.20 (0.74–1.94)	0.87	0	0.47
2014 or later	14	0.91 (0.70–1.20)	0.89	0	0.52
**Quality score**					
<7 stars	8	1.40 (0.80–2.47)	0.77	0	0.24
≥7 stars	15	0.90 (0.69–1.17)	0.97	0	0.45

*P*_s_: *P* value for heterogeneity within each subgroup; *Pt*: test for overall effect; *I*_*s*_^*2*^: *I* ^*2*^ value for heterogeneity within each subgroup.

A funnel plot, Egger’s test and a sensitivity analysis were performed to assess publication bias. The results of Egger’s test (*P* < 0.01) indicated that there was evidence of publication bias. However, the funnel plot suggested no publication bias. Additionally, the sensitivity analysis showed no publication bias, which suggested that the change in the recalculated ORs was not significant and ranged from 0.94 (95% CI = 0.73–1.19) to 1.02 (95% CI = 0.80–1.30).

## Discussion

This meta-analysis included 27 studies from 9 countries in America, Europe and Asia with 2,237 cases of MIPD and 11,854 cases of OPD. These data provided the most reliable and robust evidence to date of MIPD vs OPD. The results of the present study demonstrated that there were no significant differences between the two procedures in the POPF (grade B/C) rates, tumor size, reoperation, total cost and 5-year survival rate. MIPD was associated with decreased estimated blood loss, decreased delayed gastric emptying, increased R0 resections, reduced length of hospital stay and reduced postoperative cost but was associated with increased operative time, significantly increased operative cost and increased mortality.

Theoretically, the overall mortality in the two procedures should be similar. However, our pooled analysis showed that there was increased mortality in MIPD compared with OPD. Interestingly, the subgroup analysis showed that there were no significant differences between RLPD and OPD. Furthermore, the sensitivity analysis, which investigated the influence of a specific study on the pooled results estimate by removing one study in each turn, demonstrated that there was no significant difference between MIPD and OPD after removing the study by Adam *et al*.^19^. Additionally, a selection bias cannot be ignored in this analysis. There were no increases in mortality rates reported by high-volume centers^20^. Regrettably, the selection criteria were not shown in most of the included studies. Furthermore, MIPD is one of the most complex minimally invasive surgeries and is still a new technique with limited case reports and associated with a long learning curve^21^. Therefore, surgeons were more likely to choose an open procedure for the more advanced and larger tumors, although the pooled tumor size in MIPD vs OPD was similar. Additionally, there were few reports on first or early stage MIPD, and laparoscopic surgeons may prefer to publish the positive outcomes of MIPD. Therefore, further studies with adequate cases and controlled biases are needed to evaluate the true results of overall mortality.

The preoperative outcomes, including age, sex, BMI, ASA, tumor size and cancer diagnosis, were comparable between the different approaches. In addition, the results of the sensitivity analyses also showed that there were no significant differences. Although there was a trend towards decreased tumor size, the difference was not statistically significant. These findings suggested that the patient characteristics of the included studies were conducive to the comparison between MIPD and OPD.

The pooled overall operative time was increased in the MIPD group compared with the OPD group with high heterogeneity. Notably, the subgroup analysis showed that there was no significant difference in RLPD compared with OPD. The operative time for MIPD is closely related to the learning curve because of technical difficulties. Speicher *et al*.^21^ studied the learning curve for team-based LPD and found that although it was the biggest hurdle for the initial 10 cases of LPD, there were significant reductions in the operative time within the first 50 cases. The estimated blood loss was decreased in the MIPD group compared with the OPD group, which was also supported by the results of the sensitivity analysis. There may be a benefit associated with the magnified view that is provided by minimally invasive procedures, which resulted in a more precise resection. However, the intraoperative blood loss was a significant observation because it was a serious problem for MIPD. Therefore, surgeons may prefer an open procedure for the patients who are at risk of massive bleeding through prior evaluation, and any cases of uncontrolled bleeding during operation were converted to OPD immediately, which may have resulted in a selection bias. Thus, these results should be interpreted with caution.

The R0 resection rate and lymph node harvest are commonly used to indicate the oncologic adequacy of minimally invasive techniques^22^. The pooled analysis and sensitivity analysis showed that there was a statistically significant increase in the R0 resection rate in MIPD compared with OPD. Although our pooled analysis showed that the tumor size was similar in MIPD compared with OPD, the sensitivity analysis suggested that there were smaller tumors in the MIPD group compared with the OPD group after removing the study of Dokmak *et al*.^23^, which should be considered as a potential confounder that accounted for the higher R0 resection rates. Therefore, more evidence is needed to further explore these criteria and to evaluate these questions.

POPF rate, delayed gastric emptying and wound infection are considered to be the most unpleasant complications for pancreaticoduodenectomy, especially because POPF is a life-threatening complication. Although a high POPF rate was a serious disadvantage associated with MIPD, the results of our pooled analysis and sensitivity analysis showed that the POPF rate was similar between MIPD and OPD without heterogeneity (*P* = 0.96, I^2^ = 0%). Furthermore, the delayed gastric emptying rate was lower for MIPD than OPD without heterogeneity (*P* = 0.84, I^2^ = 0%). Additionally, a decreased wound infection rate was observed in MIPD compared with OPD.

A shorter hospital stay was another advantage of the minimally invasive techniques. This analysis indicated that MIPD may be associated with a shorter hospital stay, which was also proven by the sensitivity analysis. In other words, MIPD patients were released 3.14 days earlier than were OPD patients. Additionally, the pooled re-operative rate was similar between the two procedures. To estimate the cost burden and survival rate of MIPD compared with OPD, we examined operative, postoperative and total costs and the 5-year survival rate. The analysis showed that although the operative cost was increased, the postoperative cost was obviously decreased and there was no significant difference in total cost for MIPD compared with OPD. Furthermore, there was no significant difference in the 5-year survival rate between the two procedures. These findings indicated that MIPD may have the advantages of a shorter hospital stay without a higher re-operative rate and decreased postoperative cost without more total cost, which are closely linked to patient quality of life^3^. Langan *et al*.^24^ reported that MIPD provided a more favorable quality of life after the operation compared with OPD and that MIPD may be used as an alternative operative procedure to enhance tolerability of early adjuvant therapies, which could potentially increase survival. Croome *et al*.^3^ found that MIPD had the advantage of more rapid recovery and increased progression-free survival, which allowed patients to pursue adjuvant treatment options and eventually improved the quality of life.

### Study strengths and limitations

Our study has several strengths. One strength was the large sample size, which increased the power of the statistical analysis. We broadly and systematically reviewed multiple databases for MIPD vs OPD for studies published through February 2016 and identified all major published studies. Study selection and data extraction were performed independently and in duplicate by two investigators, which increased the validity of the results. Furthermore, studies were identified from 9 countries in the Americas, Europe and Asia, which increased the statistical generalizability. We also performed subgroup analyses for MIPD vs OPD, and these independent detailed data increased the power of the statistical analysis. Additionally, the authors of the identified studies were contacted directly to request additional data, which reduced the publication bias to some extent. It is worth mentioning that we also discussed the cost, survival rate and quality of life in MIPD vs OPD for the first time, which provided broader and more detailed evidence for the comparison.

However, the limitations of the present meta-analysis must be taken into consideration.

First, the included studies were not randomized-controlled trials, and there was only one prospective study^6^, which may have contributed to biased data. Additionally, selection bias was an important problem associated with meaningful results, which regrettably, was not shown in most of the included studies.

Second, although most of the characteristics of the included comparative studies had been adjusted for the major potential multivariable confounders, such as age, sex, ethnicity, body mass index, ASA score, year of diagnosis, tumor histology, tumor size, tumor stage and hospital type, other unmeasured confounding factors could not be ruled out. Furthermore, there were few potential confounding factors listed in several studies^19, 20, 25, 26^. Therefore, the results of this meta-analysis should be interpreted carefully because of the potential confounding factors, and further studies that are well-adjusted for confounds are needed.

Third, the quality of several of the included studies was not high despite meeting the eligibility criteria, and the sample size regarding our topic was not large^27–29^; however, the subgroup analyses addressed these issues. Several data that did not have corresponding standard deviations (SD) were excluded. Therefore, well-designed studies with more detailed evidence are needed. Additionally, there is partly overlap between the study populations of Adam *et al*.^19^ and Sharpe *et al*.^20^. We try to contract the authors for detailed data but there has been no reply so far. Furthermore, both studies were included separately in the analyses of intraoperative outcomes and postoperative outcomes. Although both studies were included in the analyses for age and tumor size, the sensitivity analyses showed that the change in the recalculated ORs was not significant. All these results indicated that although there may be a part of the data included twice, we selected them objectively and prudently in our analyses and our results were unaffected by the partly repeated data.

Fourth, although MIPD was divided into LPD and RLPD, there was not a more detailed subgroup analysis of the type of LPD, including total laparoscopic pancreaticoduodenectomy, laparoscopic pancreaticoduodenectomy, and laparoscopic pylorus-preserving pancreaticoduodenectomy. Therefore, there may be some bias influencing the reliability of the results.

Fifth, the studies included in the analysis of costs and the 5-year survival rate were limited, and the corresponding results should be used carefully.

Sixth, because the results of this meta-analysis are only based on pancreaticoduodenectomies, the available data should not be used carelessly to analyze other minimally invasive surgeries especially pancreatectomies and other pancreatic surgeries.

## Conclusions

In summary, our findings suggested that MIPD can be a reasonable alternative to OPD with the advantages of decreased estimated blood loss, increased R0 resection rate, decreased delayed gastric emptying rate, decreased wound infection and shorter hospital stay. However, MIPD should be performed at high-volume centers and more randomized-controlled trials are needed to evaluate the efficacy and appropriate indications of MIPD.

## Electronic supplementary material

Supplementary information
