# Supplementary material for: A systemic review and an updated meta-analysis: minimally invasive vs open pancreaticoduodenectomy
Source: Sci Rep. 2017 May 22;7:2220. doi: 10.1038/s41598-017-02488-4 (PMC5440387; doi:10.1038/s41598-017-02488-4)
Supplement: Supplementary file 1 — Supplementary information [file 41598_2017_2488_MOESM1_ESM.doc]

**A systemic review and an updated meta-analysis: minimally invasive vs open pancreaticoduodenectomy**

Zhanwei Zhao^1a^, Zifang Yin ^2a^, Zhenning Hang^1a^, Gang Ji^1^, Quanxin Feng^1^, Qingchuan Zhao^1^*

^1^Xijing Hospital of Digestive Diseases, the Fourth Military Medical University, 127 Changle Western Road, Xi'an, China. ^2^Shaanxi Maternal and Child Health Hospital, Xi'an, China. ^a^These authors contributed equally to this work. *Corresponding author. E-mail address: zhaoqc@fmmu.edu.cn.

**Figure 1.** Flowchart of the process for the identification of relevant studies.


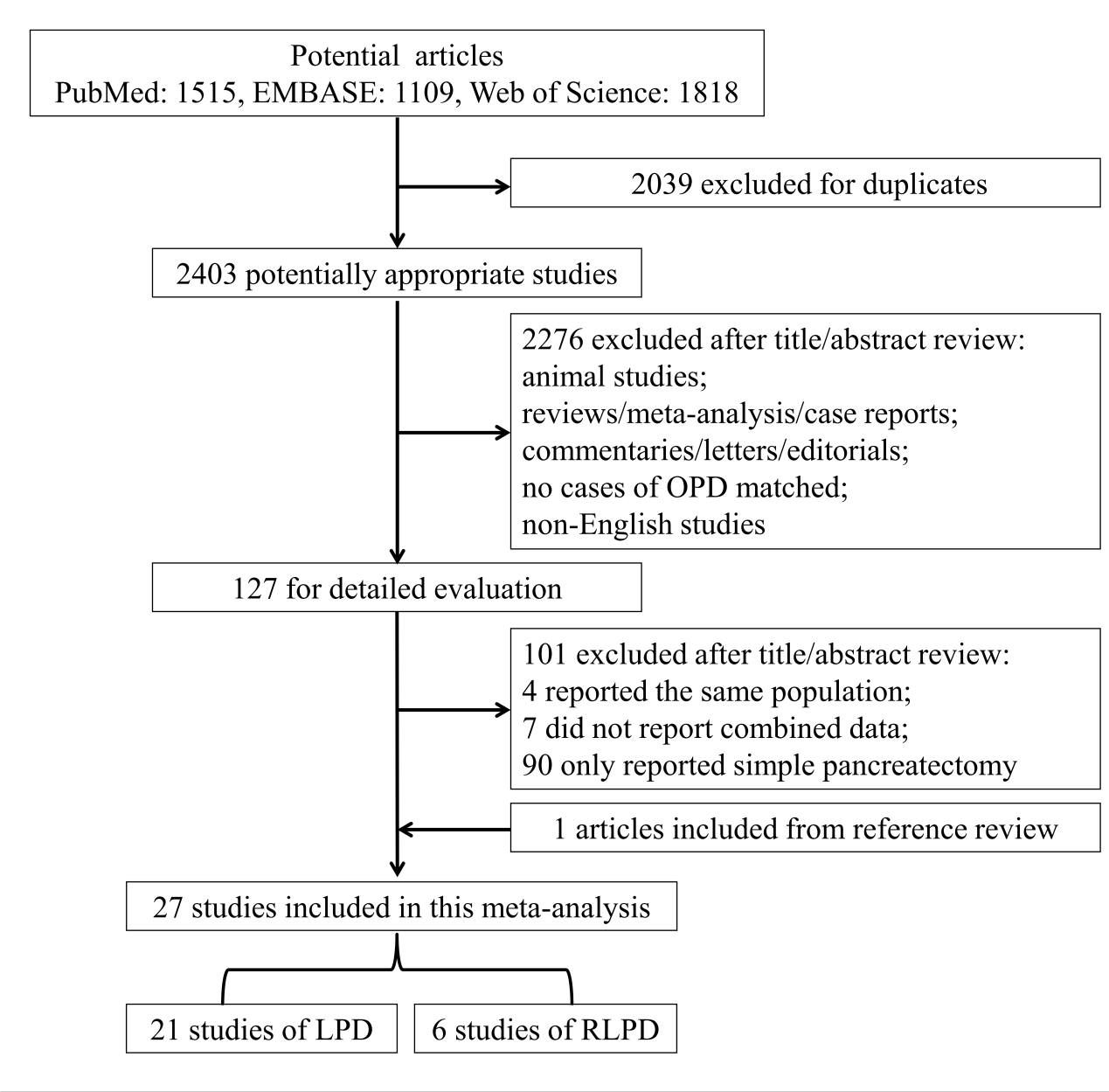


**Figure 2.** Forest plots of preoperative outcomes (random-effects models). **A**: Age. **B**: Sex (male). **C**: BMI. **D**: Tumor size. **E**: Cancer diagnosis.

**A**

**B**

**C**

**D**

**E**

**Figure 3.** Forest plots of intraoperative outcomes (random-effects models). **A**: Operative time. **B**: Estimated blood loss. **C**: R0. **D**: Lymph node harvest.

**A**

**B**

**C**

**D**

**Figure 4.** Forest plots of postoperative outcomes (random-effects models). **A**: Mortality. **B**: POPF. **C**: delayed gastric emptying. **D**: Wound infection. **E**: length of hospital stay. **F**: Re-operation.

**A**

**B**

**C**

**D**

**E**

**F**

**Figure 5.** Forest plots of postoperative outcomes (random-effects models). **A**: Operative cost. **B**: Postoperative cost. **C**: Total cost. **D**: 5-year survival rate.

**A**

**B**

**C**

**D**

**Figure 6.** Funnel plots evaluating publication bias of studies of clinically relevant postoperative pancreatic fistula. SE: standard error; OR: odds ratio.
